# Fine mapping and identification of the fuzzless gene *GaFzl* in DPL972 (*Gossypium arboreum*)

**DOI:** 10.1007/s00122-019-03330-3

**Published:** 2019-04-02

**Authors:** Xiaoxu Feng, Hailiang Cheng, Dongyun Zuo, Youping Zhang, Qiaolian Wang, Ke Liu, Javaria Ashraf, Qiuhong Yang, Simin Li, Xiaoqin Chen, Guoli Song

**Affiliations:** 1 State Key Laboratory of Cotton Biology, Institute of Cotton Research, Chinese Academy of Agricultural Sciences, Anyang, 455000 Henan China; 2 Plant Genetics, Gembloux Agro Bio Tech, University of Liège, Gembloux, Belgium

## Abstract

**Key message:**

The fuzzless gene *GaFzl* was fine mapped to a 70-kb region containing a *GIR1* gene, Cotton_A_11941, responsible for the fuzzless trait in *Gossypium arboreum* DPL972.

**Abstract:**

Cotton fiber is the most important natural textile resource. The fuzzless mutant DPL972 (*Gossypium arboreum*) provides a useful germplasm resource to explore the molecular mechanism underlying fiber and fuzz initiation and development. In our previous research, the fuzzless gene in DPL972 was identified as a single dominant gene and named *GaFzl*. In the present study, we fine mapped this gene using F_2_ and BC_1_ populations. By combining traditional map-based cloning and next-generation sequencing, we mapped *GaFzl* to a 70-kb region containing seven annotated genes. RNA-Sequencing and re-sequencing analysis narrowed these candidates to two differentially expressed genes, Cotton_A_11941 and Cotton_A_11942. Sequence alignment uncovered no variation in coding or promoter regions of Cotton_A_11942 between DPL971 and DPL972, whereas two single-base mutations in the promoter region and a TTG insertion in the coding region were detected in Cotton_A_11941 in DPL972. Cotton_A_11941 encoding a homologous gene of *GIR1 (GLABRA2*-*interacting repressor)* in *Arabidopsis thaliana* is thus the candidate gene most likely responsible for the fuzzless trait in DPL972. Our findings should lead to a better understanding of cotton fuzz formation, thereby accelerating marker-assisted selection during cotton breeding.

**Electronic supplementary material:**

The online version of this article (10.1007/s00122-019-03330-3) contains supplementary material, which is available to authorized users.

## Introduction

In the history of development economics, cotton has been thought as a great commercial crop due to its edible oil and especially fiber which regarded as an important natural textile resource. Similar to trichomes in Arabidopsis, cotton fibers are single cells derived from the ovule epidermis (Ishida et al. 2008; Wan et al. 2014). Cotton fiber is categorized into two types according to the initiation stage: lint, developing before or at 0 days post-anthesis (DPA), and fuzz, developing approximately 3–5 DPA (Lee et al. 2007). Compared with lint, fuzz impedes seed germination and therefore leads to cause a low fiber production in the next growing season. Fuzzless breeds are thus increasingly being considered as important resources in cotton genetics and breeding.

Fuzzless mutants have been a major focus of cotton research since 1927 (Jiang et al. 2015; Liang et al. 2015). Two fuzzless loci in *G. hirsutum*, N_1_ and n_2_, are currently known to inhibit fuzz development. N_1_ is a single dominant gene and controls a fuzzless–lint trait (Rong et al. 2005; Wan et al. 2014, 2016). According to map-based cloning, N_1_ is located on chromosomes 12 (A12) and has been annotated as a MIXTA-like gene, *MYB25*-*like* (also known as *GhMML3*). In the mutant N_1_, *GhMML3* also shows reverse transcriptional activity and generates natural antisense transcripts (NATs) (Wan et al. 2016). The bidirectional transcripts of *GhMML3_A12* form double-stranded RNAs and small RNAs. Small RNAs may mediate self-degradation and self-cleavage of mRNA and then result in a naked seed phenotype. In addition, data from virus-induced gene silencing (VIGS) suggest that disruption of *GhMML3* in TM-1 leads to a fuzzless or reduced fuzz phenotype. The other fuzzless gene, *n*_*2*_, is a single recessive locus, located on chromosome26 (D12) (Zhu et al. 2018). But the candidate gene for *n*_*2*_ has not yet been identified. Recent research has shown that *N*_*2*_ and *Li*_*3*_ are, respectively, responsible for the production of fuzz fiber and lint fiber in the fuzzless–lintless mutant XuZhou142 (Wu et al. 2018). Using a map-based cloning strategy, the authors of cited study identified *Li*_*3*_ as another MML transcription factor (*GhMML4_D12*) regulating lint development. Nevertheless, the *n*_*2*_ gene has remained elusive. The inheritance of fuzz and lint fiber initiation and development is complex (Naoumkina et al. 2016; Sun et al. 2017). These studies on fuzzless mutants and fiber-related genes have contributed to our understanding of the mechanism of fiber/fuzz initiation and regulation.

In contrast to research on N_1_ and n_2_, few systematic studies have been carried out on the diploid *G. arboreum* fuzzless mutant DPL972. A comparison of molecular mechanisms between diploids and tetraploids is thus needed (Parekh et al. 2016). DPL972 is a near-isogenic line (NIL) of the wild-type DPL971. They carry the same genetic background with the exception of traits for fuzz and flower color. In a whole-genome expression analysis, *MYB23*, *MYB5* and *TTG1* were significantly differentially expressed between DPL971 and DPL972. Wang et al. (2013) subsequently identified *MYB23* as a homologous gene of *GLABRA1* involved in the regulation of Arabidopsis trichome development and cotton fiber initiation. Whether *MYB23* controls the fuzzless trait in the mutant DPL972 is still unknown. In a previous study, we developed SSR markers linked to *GaFzl* and mapped this gene to chromosome A08 (Feng et al. 2016), but were unable to identify the exact gene that controls fuzz development.

In the present study, we explored and identified *GaFzl* to better understand fiber initiation and fuzz development. To fine map the target gene, we integrated bulked segregant analysis sequencing (BSA-Seq), map-based cloning and RNA-Sequencing (RNA-Seq) strategies. We found a *GIR1*gene significantly differentially expressed between the mutant and wild type in the candidate region. Our finding should contribute to a deeper understanding of fuzz development.

## Materials and methods

### Materials and population construction

In this study, we used *G. arboreum* DPL971 and its fuzzless mutant DPL972, which had been self-pollinated for more than 10 generations in our laboratory. Plants were grown annually in accordance with standard agronomic practices at the farm of the Institute of Cotton Research, Chinese Academy of Agricultural Sciences, Anyang, China.

To fine map the target gene, we consecutively constructed F_2_ and BC_1_ populations. The F_2_ population comprised 4010 individuals derived from a cross between DPL971 and DPL972 in 2014. F_1_ plants were then backcrossed with DPL971 in 2017 to produce a BC_1_ population comprising 607 individuals. These two populations were used to fine map the fuzzless gene in DPL972. The F_2_ population was also used as a source of individuals with extreme traits for construction of sequencing pools. The phenotypic segregation ratios of the two populations were analyzed by a *χ*^2^ goodness-of-fit test in SAS.

### Genomic DNA and RNA extraction

Young leaves from the parents and F_2_ and BC_1_ individuals were collected and stored at − 80 °C. Young leaf tissues were frozen in liquid nitrogen and ground into a fine powder using a hybrid grinding machine (Retsch MM400, Verder Scientific, Germany). Genomic DNA was then extracted using the cetyltrimethylammonium bromide method and stored at − 20 °C.

Fiber-bearing ovules were excised from developing cotton bolls at 2-day collecting intervals [i.e., at 1, 3 and 5 days post-anthesis (DPA)]. The ovule samples were wrapped in tinfoil, frozen directly in liquid nitrogen and stored at − 80 °C for subsequent experiments. Total RNA was isolated from the collected ovules using an EASY-spin Plus Plant RNA kit (Aidlab, China) and an RNA Prep Pure Plant kit (Polysaccharides and Polyphenolics-rich) (Tiangen, China) according to the kit manuals. Total RNA integrity, purity and concentration were examined using an Agilent 2100 RNA 6000 nanokit (Agilent Technologies, Santa Clara, CA, USA). Suitable RNA samples (A260/A280 ratio = 1.8–2.0, A260/A230 ratio > 1.5, and RNA integrity number > 8) were stored at − 80 °C for subsequent deep sequencing and quantitative real-time PCR (qRT-PCR) analysis.

### BSA-Seq and re-sequencing analyses

Genomic DNA of 30 extremely fuzzy and 30 extremely fuzzless individuals from the F2 progenies was selected to generate two bulked pools. The DNAs of parents DPL971 and DPL972 were also extracted for BSA-Seq and re-sequencing on an Illumina NovaSeq 6000 platform. Raw reads from the four DNA pools were filtered and then aligned to the cotton genome sequence using the Burrows–Wheeler alignment tool (BWA) (Li and Durbin 2009). GATK and Picard were used to detect InDels and single-nucleotide polymorphisms (SNPs) (Mckenna et al. 2010). Euclidean distance (ED) and ΔSNP index values were calculated to identify candidate genomic regions associated with the fuzzy trait (Fekih et al. 2013). In regards to the ED strategy, ED values of regions other than target gene-related ones tend to be consistent and trend toward 0. ΔSNP index values were determined as the difference in the SNP index between fuzzy and fuzzless pools with values of genomic regions including the target gene expected to approach 1. By examining ED and ΔSNP index values between the two bulked pools, the plot peak regions above the threshold value were defined as candidate regions for association with the fuzzless trait.

### Fine mapping of the fuzzless gene

In our previous study, we developed genome-wide SSR markers and identified 13 markers linked to the fuzzless gene. In the present study, we used these same markers to further fine map the target gene. To perform the subsequent mapping, additional SSR and InDel marker primers were designed with software SnapGene and Primer5 software based on the results of re-sequencing. Those markers with suitable levels of polymorphism and high specificity between the two mapping parents and bulked pools were used to construct the linkage map and narrow the candidate region.

### RNA-Seq analysis

To explore the molecular mechanism underlying fuzz initiation and development in *G. arboreum*, fiber-bearing ovules were selected at three developmental stages (1, 3 and 5 DPA). Two biological replicates of three stages of ovules of the DPL972 fuzzless mutant and its wild-type DPL971 were used to create 12 independent RNA libraries. These libraries were subjected to 101-cycle paired-end sequencing on an Illumina HiSeq 4000 platform at Berry Genomics (Beijing, China).

Raw data from the 12 sample libraries were assessed and filtered to remove adaptors and low-quality reads. The remaining high-quality reads, referred to as clean data, were aligned to the reference genomes of *G. arboreum* (Li et al. 2014), *G. raimondii* (Wang et al. 2012) and *G. hirsutum* (Li et al. 2015; Zhang et al. 2015a) using TopHat2 (Kim et al. 2013). The mapped sequences were then annotated against the above-mentioned reference genomes, with a maximum of two bases allowed per mismatch in our alignments. In addition, the number of fragments per kilobase of transcript per million mapped reads (FPKM) of each transcript in the fuzzless mutant DPL972 and wild-type DPL971 was calculated using Cuffdiff (v2.2.1) software (Trapnell et al. 2016). Based on these FPKM values, a false discovery rate (FDR) < 0.01 and |log2 (ratio)| ≥ 1 were set as criteria to identify differentially expressed transcripts between the mutant and the wild type. *P* values of the statistical *t* test were adjusted and applied to control FDR using multiple testing procedures. HemI 1.0 was used for clustering analysis of DEGs (Deng et al. 2014).

### Gene cloning and multiple sequence alignment

Gene- and promoter-amplification primers were designed using Primer5 and SnapGene software. The full-length sequences of genes and promoters were, respectively, amplified using cDNA and genomic DNA from DPL971 and DPL972. PCR products were purified using a QIAquick Gel Extraction kit and sequenced by Sangon Biotech (Shanghai, China). The resulting sequences were aligned with DNAMAN software.

### QRT-PCR

To verify the accuracy of RNA-Seq and identify expression levels of candidate genes, qRT-PCR was also performed. Fiber-bearing ovules were selected at two developmental stages (1 and 3 DPA). RNA samples (1–2 µg) were subjected to reverse transcription using TransScript All-in-one First-strand cDNA Synthesis SuperMix (TransGen Biotech). QRT-PCR amplifications were conducted using TransStart TOP Green qPCR SuperMix (TransGen Biotech) on an ABI Prism7500 Fast Real-time PCR System (Applied Biosystems, USA). DEG expressions were normalized using *GaHis3* as a reference gene. Primers for amplifying DEGs were designed with NCBI Primer-BLAST (http://www.ncbi.nlm.nih.gov/tools/primer-blast/) and BatchPrimer3 (http://wheat.pw.usda.gov/demos/BatchPrimer3/) and synthesized by Genewiz (Beijing, China). Primer sequences are provided in Table S1. Each qRT-PCR reaction included three biological replicates and three technical replicates. Relative expression levels were calculated using the $2^{{ - {\Delta \Delta }C_{\text{t}} }}$ method (Livak and Schmittgen 2001).

## Results

### Phenotypes and segregation analysis of genetic populations

**Fig. 1:**
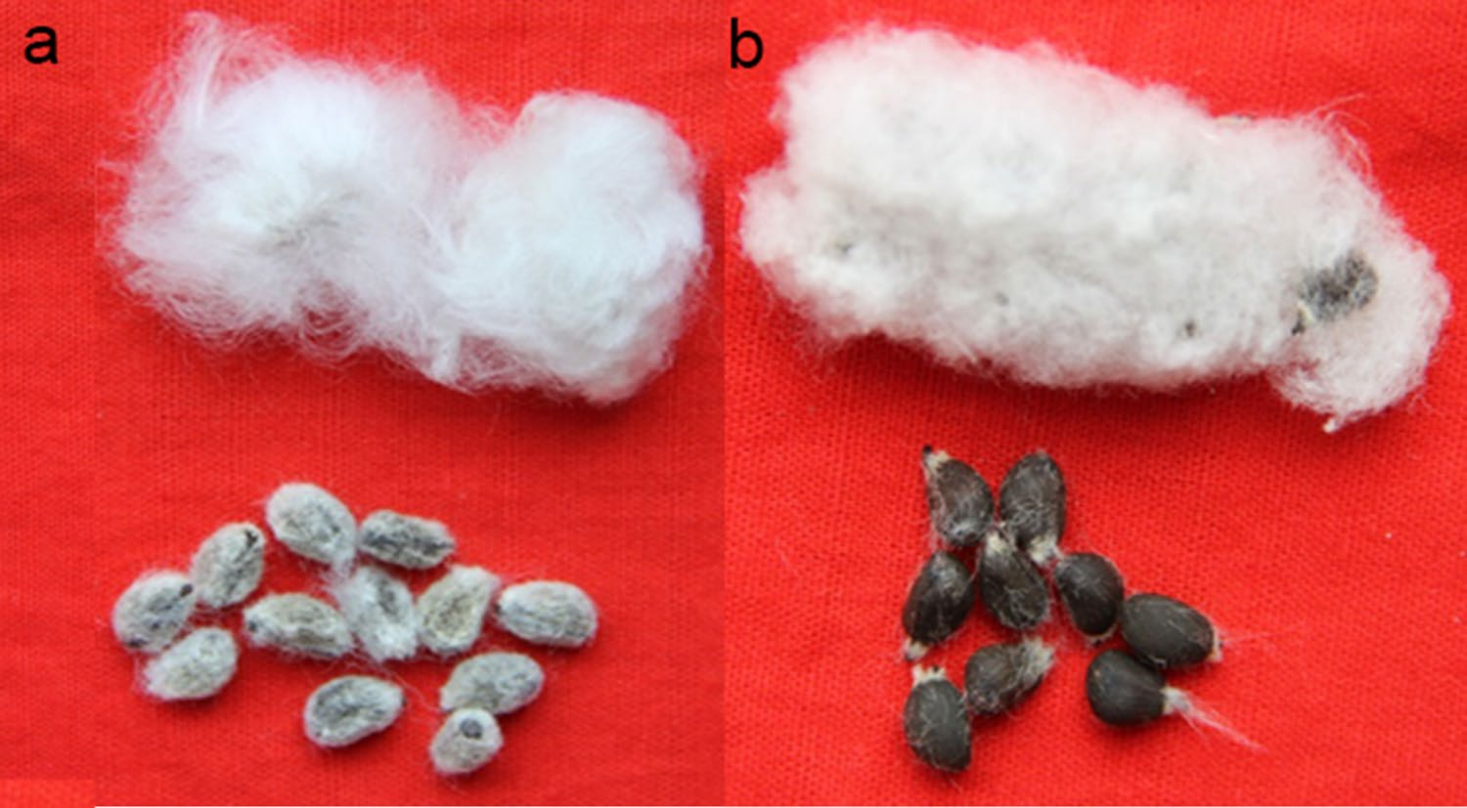
Phenotypes of fuzzy seeds DPL971 (**a**) and the fuzzless mutant DPL972 (**b**)

In the current study, we used two mapping parents: wild-type DPL971 and the fuzzless mutant DPL972 (Fig. 1). We successively constructed F_2_ and BC_1_ populations in 2014 and 2016. Phenotypes of F_1_ individuals derived from the cross between DPL971 and DPL972 were always consistent with those of DPL972, which indicates that the fuzzless trait in *G. arboreum* was dominantly inherited. As can be seen from Table 1, the observed segregations in F_2_ and BC_1_ generations fit the expected phenotypic segregation ratios of 3:1 and 1:1, respectively. Consistent with our previously published data (Feng et al. 2016), the phenotypes of the BC_1_ population further validated our finding that a single dominant gene controls the fuzzless phenotype in DPL972. The new BC_1_ population and the previous F_2_ were both used for subsequent mapping of *GaFzl.*

**Table 1:** Genetic analysis of fiber trait in parents and two segregating populations

Material	Total	Phenotype		Observed ratio	Expected ratio	*χ* ^*2*^	*P*
		Fuzzless	Fuzzy				
DPL971	20	0	20				
DPL972	20	20	0				
F_1_	300	300	0				
F_2_ population	4010	3004	1006	2.99:1	3:1	0.02	0.9
BC_1_ population	607	315	292	1.08:1	1:1	0.87	0.35

### Mapping of *GaFzl* gene to chromosome A08 by BSA-Seq

**Fig. 2:**
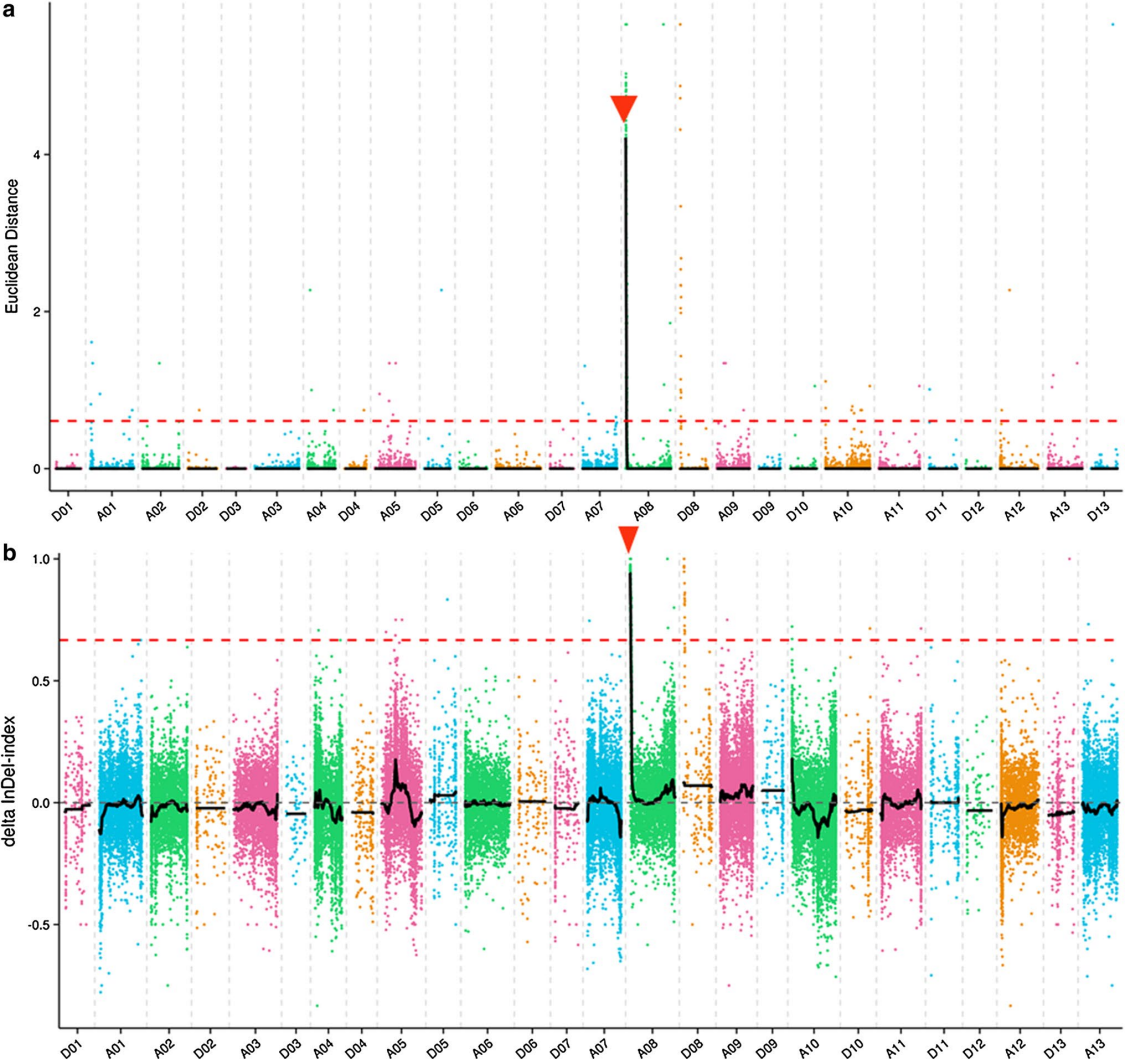
Candidate region of *GaFzl* based on application of a combined ED/ΔSNP index strategy to InDels. **a** ED graph of InDels between DPL971, DPL972 and two bulked pools. **b** ΔSNP index graph of InDels between DPL971, DPL972 and two bulked pools

**Fig. 3:**
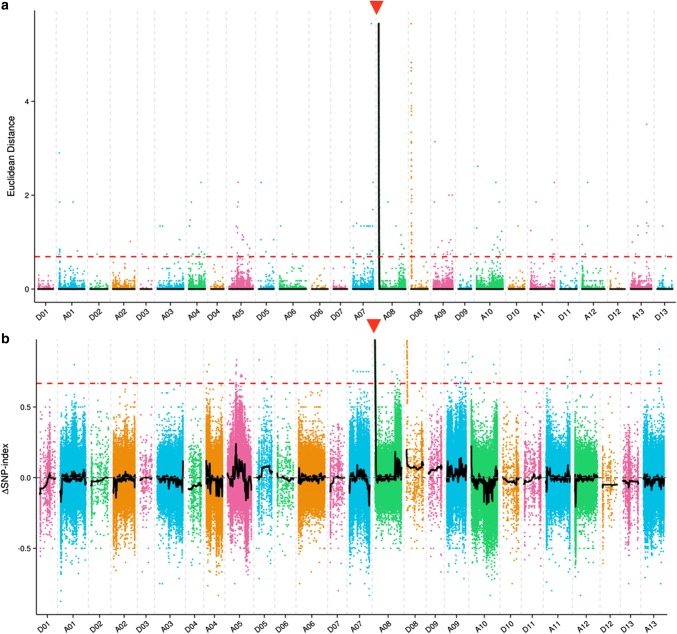
Candidate region of *GaFzl* based on application of a combined ED/ΔSNP index strategy to SNPs. **a** ED graph of SNPs between DPL971, DPL972 and two bulked pools. **b** ΔSNP index graph of SNPs between DPL971, DPL972 and two bulked pools

To rapidly map and identify the genomic region contributing to the fuzzless trait in G *arboreum* DPL972, we used BSA-Seq to fine map the mutation. A total of 228.9 Gb clean data were obtained by re-sequencing. These re-sequencing reads were then aligned to the preferred TM-1 reference genome using BWA software (Table S1). Details of all SNPs and InDels between DPL971 and DPL972 as well as the two bulked pools were listed in Tables S2 and S3. A total of 353,611 high-quality SNPs and 51,244 small InDels were filtered out and identified between the two bulked pools. Applying the ED method to InDels and SNPs, we chose 0.35 (median + 3SD of all fitted values of points) as the threshold value and identified a 2.23 Mb region from 76,294 to 2,305,623 bp on chromosome A08 (Figs. 2a, 3a). Using the ΔSNP index strategy, we also identified a 2.22 Mb region from 68,670 to 2,195,304 bp on A08 (Figs. 2b, 3b). These regions overlapped. The merged region from 76,294 to 2,195,304 bp is thus likely the candidate region of the *GaFzl* locus controlling the fuzzless trait in *G. arboreum*.

### Narrowing of the *GaFzl* gene to a 70-kb region using SSR and InDel markers

In our previous study, we identified 62 polymorphic SSR markers between DPL971 and DPL972 by screening the whole genome. Only one marker on chromosome A08 showed a tight linkage with the fuzzless trait. Because the exact region was undetermined, we developed 652 SSR markers on A08, of which 13 polymorphic SSR markers showed a linkage relationship with the fuzzless trait (Table 2). The location of these SSR markers overlapped with the region obtained by BSA-Seq.

**Table 2:** Details of linked markers on A08 used for fine mapping

Primer name	ForwardPrimer (5′ → 3′)	ReversePrimer (5′ → 3′)	Product Length (bp)	Start position (bp)
InDel5	CGAATCCTGAACCCCAAACCTAAAC	CATCACCATGGCAACAACTCC	376	532,619
InDel6	CGCGAGGACTAAAATTTGAAAGTTTGGA	GGTAAGGATTGGGGCATTAACTGG	178	534,601
SSR82	CCTTCCATGCATATTGGAAA	CAAAGCACCCAATTTCAAGG	283	580,404
InDel9	GATTTCCCTTTCATCAAATATTCTATGTTAGCG	ACTCAAATACTTAGTATACCATATTAGCCTCTTTC	536	650,514
SSR3590	AAACCCAATATATTCTGAGTTAAATGT	TCACTATAACTAGCGGGTGGAAA	184	665,115
SSR3616	GGGATACCTGCAAACATTGTG	TTCATGGCCTTCCTCTCTGT	154	742,113
SSR3668	TGGATCGGTAATGGTAGAAAGC	AGCAAGGTCTTAGATGGCAA	240	773,922
SSR3692	GCTGTGAGGACATGAAACGA	TTGGTCTCCCTTTAGCAACG	222	821,505
SSR3699	TCGAGTTCGGTTAACTCATAACA	CCGAACACAAACTTAATTGGAA	151	877,473
SSR9	AAATATAACGATGTGGGTGGAAA	TGTCATGACTTAACCGAACACA	292	877,601
SSR6-38	CGTCGTTGGGTACTGATCCA	CCAACCAAGCCTTTCACACG	329	1,006,357
SSR67	AAGTGGGATATTGCCATCCA	GCTATGTTAATAGTGTCATCAAATGAA	274	1,083,921
SSR3741	AACATGGTCAAGATAATTGCACTA	AAAGTGCATACAGATGCCAAA	244	1,091,420
SSR3465	GCTTAGGACGGATTTGGTAAA	TGCAAGTTTGAAGGAATATAATGAA	229	1,478,980
SSR3257	GAATACTCCCTCATCCCAATAAA	TGATCGACACTTCTTCTGTCTCA	246	1,578,266
SSR3291	ATCCTTGTTATGCTCCGCTC	TTCAATGGACTGTGAGGGTAAA	236	1,683,443
SSR3370	TTTGGATCGGATTTGGGTTA	GCAATCAAATCCTTGAAGCC	188	2,183,521

**Fig. 4:**
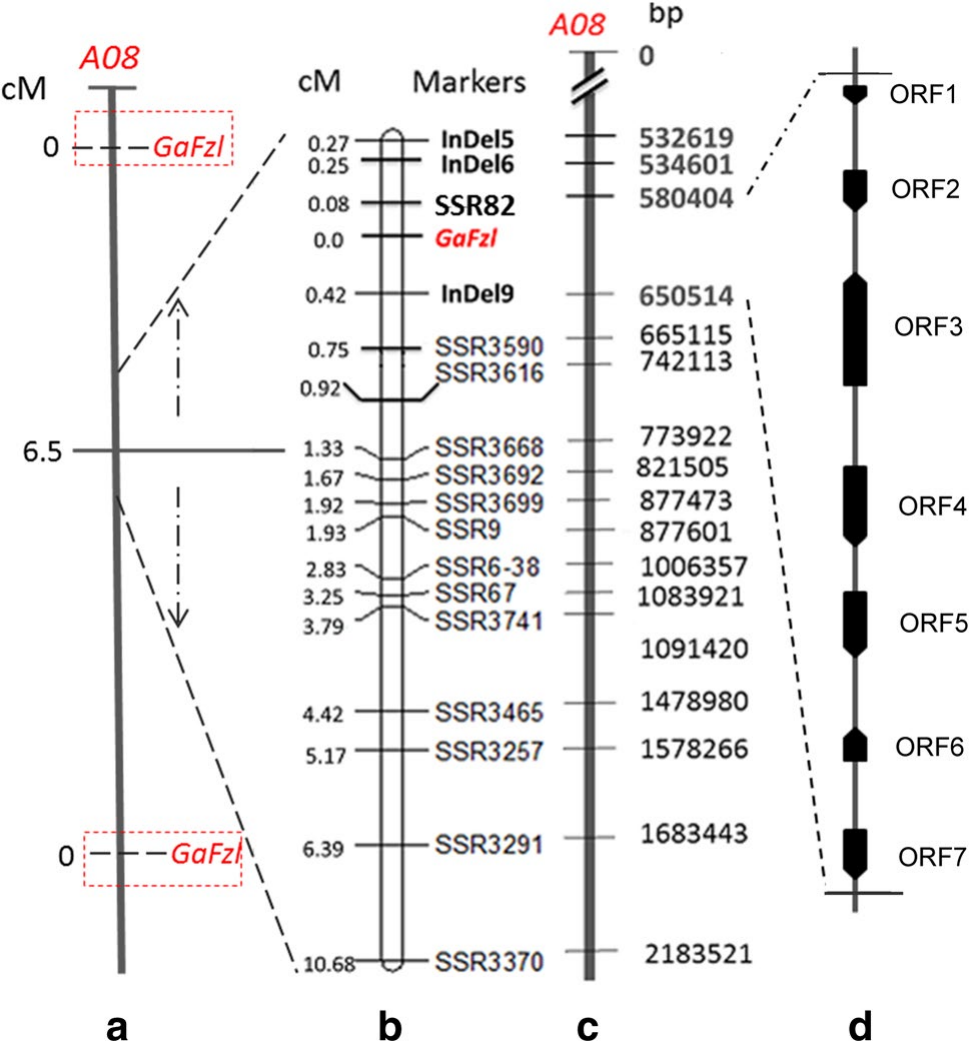
Map position of the fuzzless gene *GaFzl* in *Gossypium arboreum* DPL972 on chromosome A08. **a** Primary mapping for the fuzzless trait. Map distances (cM) are on the left. **b** Linkage map based on SSR and InDel markers. Map distances (cM) are on the left with marker names on the right. **c** A physical map of the candidate region for the *GaFzl* gene. Numbers on the right indicate loci of the markers. **d** ORFs in the candidate region

To rapidly shorten the physical mapping interval and validate the target gene, we developed 11 InDels and 98 SSR markers (detailed in Tables 2 and S4) based on the results of the re-sequencing of DPL971, DPL972 and the bulked pools. *GaFzl* was narrowed down to a 70-kb region between SSR82 and InDel9 on chromosome A08. According to cotton genome annotation information, only seven ORFs exist in the corresponding genomic region (Fig. 4). Details on ORF position are given in Table 3.

**Table 3:** Position details of seven ORFs

Gene	Start (bp)	End (bp)	Strand	CDS length (bp)	Annotation
ORF1	580,856	581,116	**+**	261	*GLABRA2* interacting repressor
ORF2	605,735	606,576	**+**	813	Probable CCR4-associated factor 1 homolog 11
ORF3	607,704	618,632	**–**	2865	Serine/threonine-protein phosphatase BSL2
ORF4	626,811	632,509	**+**	1770	Outer envelope protein 61
ORF5	635,236	638,304	**+**	1158	E3 ubiquitin-protein ligase RHF2A
ORF6	639,890	642,641	**–**	441	Succinate dehydrogenase subunit 6, mitochondrial
ORF7	645,188	647,657	**+**	651	Small heat-shock protein 1

### Application of RNA-Seq to filter DEGs

**Fig. 5:**
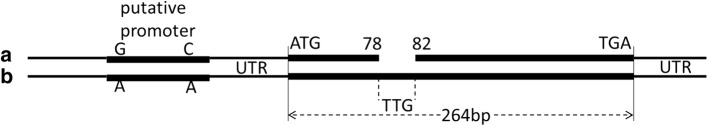
Differentially expressed genes detected by RNA-Seq. **a** Venn graph of DEGs during different ovule developmental stages. **b** Numbers of up-regulated/down-regulated DEGs at different stages

To further identify the candidate gene related to fiber initiation and fuzz development, RNA-Seq was performed on 12 independent ovule RNA libraries derived from two replicates each of the fuzzless mutant DPL972 and WT DPL971 at three fiber developmental stages (1, 3 and 5 DPA). After removal of adaptors and low-quality reads, 151,811,659 and 149,115,836 clean reads (44.73 and 45.54 Gb of clean data) were generated from DPL971 and DPL972, respectively. On average, we obtained 25,077,291 high-quality reads (7.52 Gb of clean data) from each library (Table S5). More than 91.00% of the total reads were then aligned and mapped to the published *G. arboreum* genome. After calculating FPKM values, a FDR normalized to a *P* value < 0.01 and a |log2 (ratio)| ≥ 1 was set as appropriate thresholds to distinguish significant differences in gene expression. The numbers of DEGs identified by RNA-Seq are statistically summarized in Fig. 5a as a Venn diagram. A total of 383 DEGs were identified as differentially expressed between the fuzzless mutant DPL972 and the wild-type DPL971 at three developmental stages (Fig. 5b). Of them, 370 were differentially expressed at 1 DPA, while 13 and 22 were detected at 3 DPA and 5 DPA, respectively. Although fuzz emerged from the ovule epidermis at 3–5 DPA, this result indicates that the genes controlling fuzz development may come into play at an earlier stage.

### Identification and sequence analysis of candidate genes

According to the genome comparison and RNA-Seq data annotation, only two DEGs (ORF1 and ORF2) were tandemly located in the mapping region and both were notably up-regulated in DPL972 at 1 DPA. The other five genes exhibited no differences in expression levels. In addition, no InDels or SNPs were observed in the target region that could lead to a point mutation or frame-shift mutation of those five genes based on the re-sequencing results. We therefore finally identified ORF1 and ORF2 as fuzzless trait candidate genes. ORF2 (Cotton_A_11942), annotated as *CAF1*, encodes a transcription factor annotated as a component of the *CCR4* complex. In contrast, no annotation information is available in cotton for ORF1 (Cotton_A_11941), it could only be annotated on the basis of sequence similarity as a *GIR1* gene (*AT5G06270*), which interacts with the *GLABRA2 (GL2)* repressor and is a regulator of root hair development.

**Fig. 6:**
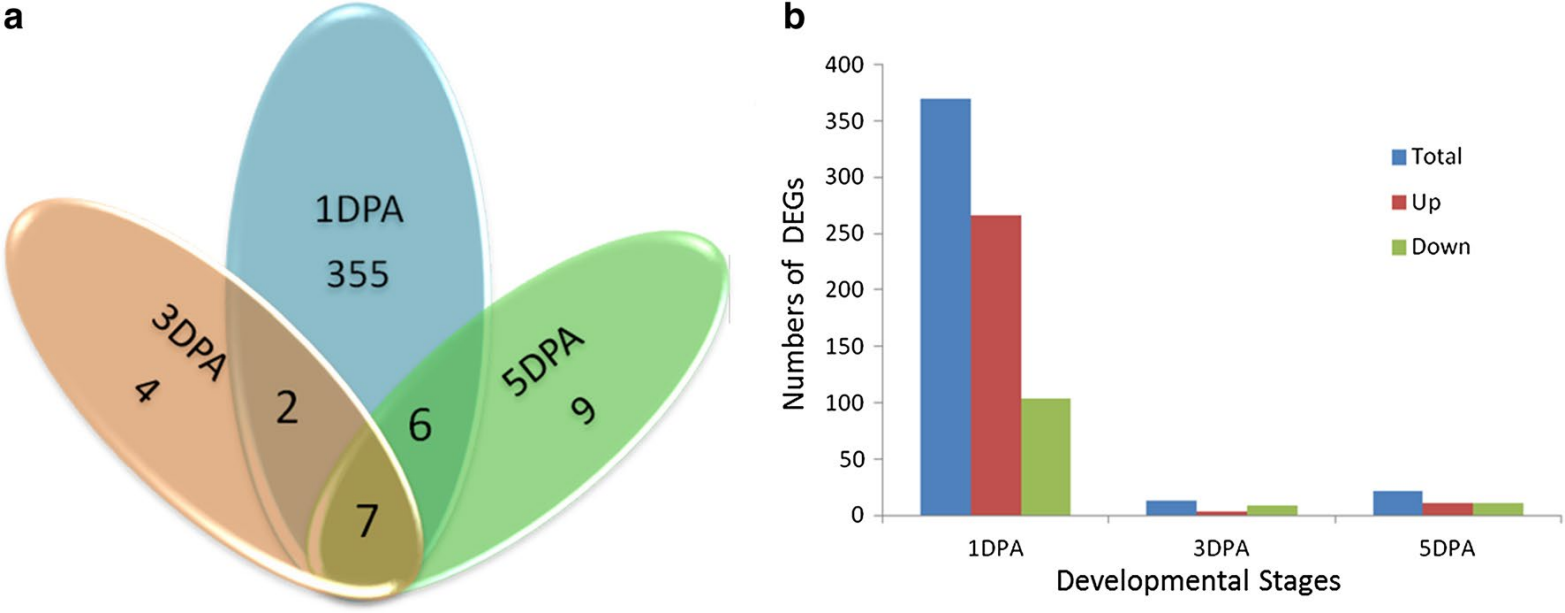
Sequence comparison of Cotton_A_11941 between DPL971 and DPL972. **a** Gene sequence in DPL971. **b** Gene sequence in DPL972

Based on the re-sequencing data and amplification results, we compared the sequences of Cotton_A_11941 and Cotton_A_11942 in DPL971 and DPL972. No differences in the genomic or promoter sequence of Cotton_A_11942 were observed between the two parents. In Cotton_A_11941 of DPL972, however, we detected a TTG insertion that resulted in the insertion of a leucine rather than causing a shift or nonsense mutation (Fig. 6). In addition, BSA and re-sequencing data from the two parents and bulked pools revealed single G → A and C → A mutations in the promoter region of Cotton_A_11941 (Fig. 6). These mutations in the promoter may lead to differences in expression levels. Cotton_A_11941 is thus the most likely gene corresponding to *GaFzl*.

### Expression profiling to check candidate genes

**Fig. 7:**
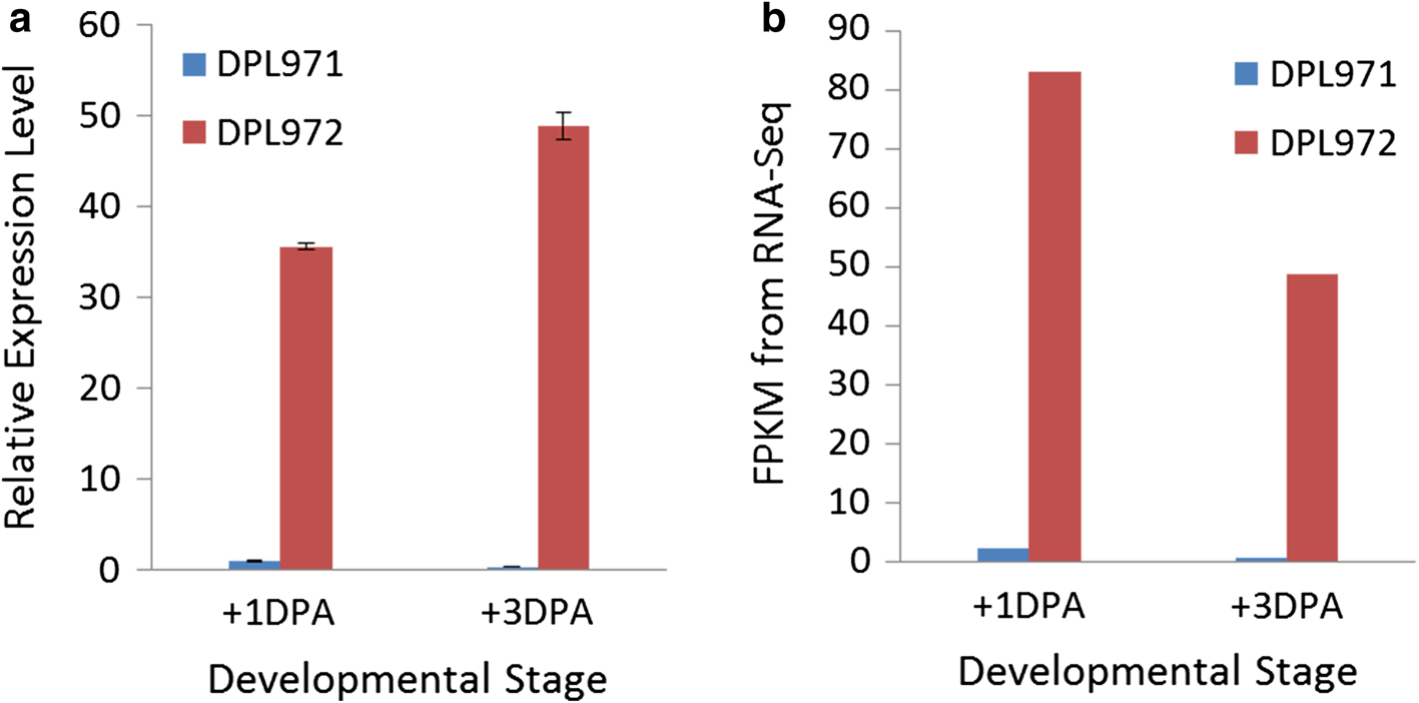
Expressions profiling of Cotton_A_11941 in DPL971 and DPL972. **a** Relative expression level. **b** FPKMs from RNA-Seq data. The x-axis represents different developmental stages. The y-axis corresponds to relative expression level. Error bars indicate standard deviations

To verify the authenticity of the RNA-Seq results, we examined the expression levels of candidate genes in ovules at different stages by qRT-PCR. As shown in Fig. 7, expression levels of Cotton_A_11941 at 1 and 3 DPA in DPL972 were much higher than those in DPL971, consistent with the RNA-Seq data. Interestingly, Cotton_A_11941 was annotated and identified as a regulator of root and trichome development, thus indicating that elevated expression of Cotton_A_11941 is more likely to result in a repression of fuzz development. This result further suggests that Cotton_A_11941 is the candidate gene controlling fuzz development.

## Discussion

### A sequencing-assisted strategy is an efficient method for gene fine mapping

Identification of target genes based on natural or mutagenic mutants is the central idea of forward genetics (Chang et al. 2016; Andres et al. 2017; Liu et al. 2018; Qureshi et al. 2018; Yang et al. 2018). Map-based cloning, the most efficient and visual strategy for gene identification, has been used to obtain numerous genes (Dou et al. 2018; Han et al. 2018; Jeong et al. 2018; Karthikeyan et al. 2018). For instance, a previous study used this technique to map and characterize a dominant single gene *Gl*_*2*_^*e*^ that controls the development of gland pigment (Cheng et al. 2016). However, this strategy has apparent drawbacks as it is time-consuming and laborious. To guarantee accuracy and pinpoint recombinants, we always expend considerable time to develop sufficiently large populations and copious markers (Zhu et al. 2017). Otherwise, the experiment may be hindered or even failed to yield final results because variants are absent or the mapping parents possess low polymorphism.

Recently, BSA and re-sequencing have been of tremendous assistance in gene fine mapping and cloning (Yang et al. 2017). For instance, Wang et al. (2017) narrowed the genetic locus of citrus polyembryony to an 80-kb region through the strategy including de novo sequencing and BSA-Seq and identified a *CiRWP* gene responsible for this apomictic phenotype. As another example, Huang et al. (2017) performed BSA and deep sequencing in *Setaria viridis* to fine map the sparse panicle gene to a 1 Mb interval and characterized *SvAUX1* as a regulator of inflorescence development and root gravitropism. In cotton, Zhu et al. (2017) combined the strategy of BSA and VIGS strategies to rapidly map and identify the target *v1* locus, an approach that proved to be an efficient and reliable method to complete map-based gene cloning.

In the current study, our combined application of BSA-Seq and traditional fine mapping uncovered a quite very narrow candidate region of 70 kb. We also performed a RNA-Seq analysis to assist filtering of candidate genes. We eventually confirmed that only one candidate gene existed in the 70-kb region. High-throughput sequencing has greatly improved and accelerated fine mapping and cloning of genes with important agronomical traits.

### Transcription factors and other genes regulate fiber and fuzz development

As mentioned earlier, cotton fiber initiation and development share similarity with Arabidopsis trichome development (Hulskamp 2004; Yoshida et al. 2009). Previous studies have noted that homologs of the MYB-bHLH-WD40 complex also influence fiber initiation in cotton (Lu et al. 2017). *GhMYB2 (GL1)*, *GhMYB25/MIXTA*, *GhTTG1* and *GhHOX3 (GL2)* are essential for fiber initiation and development (Walford et al. 2012; Wang et al. 2014; Salih et al. 2016). In contrast, *GhCPC* functions as a negative regulator for fiber early initiation and elongation (Zhang et al. 2015b). Recently Zhang proposed the existence of a new MYB-MIXTA-WD complex, including the MIXTA and WD40 genes that controls fiber and fuzz formation.

**Fig. 8:**
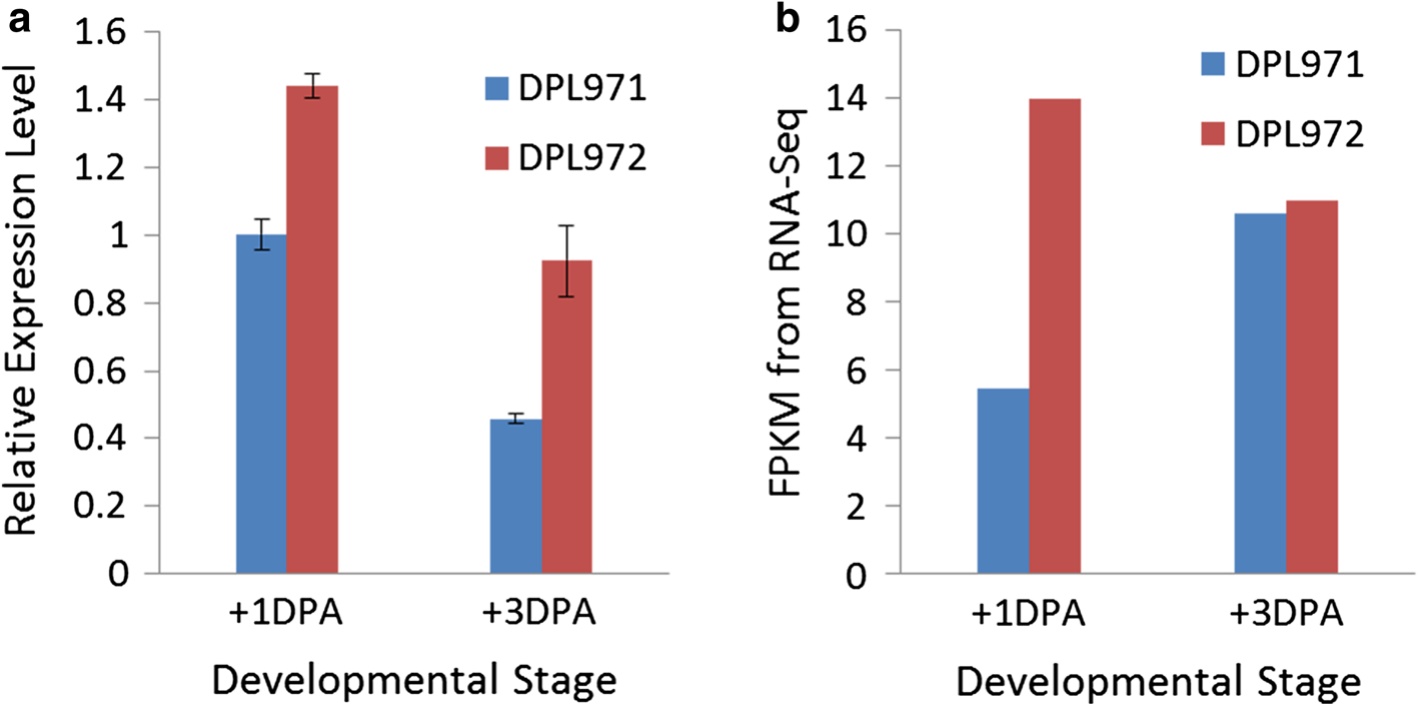
Expressions profiling of Cotton_A_11942 in DPL971 and DPL972. **a** Relative expression level. **b** FPKMs from RNA-Seq data. The x-axis represents different developmental stages. The y-axis corresponds to relative expression level. Error bars indicate standard deviations

In this study, we found that Cotton_A_11942 is differentially expressed in DPL971 and DPL972, with qRT-PCR results consistent with the RNA-Seq data (Fig. 8). This gene encodes a cinnamoyl-CoA reductase (CCR)-associated factor (*CAF1*). Previous investigations have demonstrated that high expression of *GhCCR4* in fiber can cause an obvious thickening of fiber cell wall, which results in a pre-termination and a shorter fiber length. Although we failed to detect variable sites in Cotton_A_11942 between DPL971 and DPL972, distinct expression differences were definitely observed. Cotton_A_11942 may therefore act during fiber elongation and secondary wall thickening or function downstream of genes controlling fuzz development.

We found that the most likely candidate gene, Cotton_A_11941, shared a high sequence similarity with *GIR1* in *Arabidopsis* (Wu and Citovsky 2017). Using loss- and gain-of-function strategies, Wu and Citovsky (2017) discovered that *GIR1* and *GIR2* interact with the repressor of *GL2* to repress root hair development. We checked the expression levels of *GaGL2* in our stud*y*, and detected no significant differences between the wild type and the mutant (Fig. S1), which suggest that *GaGIR1* may interact with *GaGL2* and repress the transcriptional activity of *GaGL2* to regulate the downstream gene and fuzz formation. The function of *GIR1* in cotton is still unclear, however, and we suspect that *GIR1* also influences fiber/fuzz initiation and formation. Cotton_A_11941, as the homolog of *GIR1,* is thus most likely the gene that regulates fuzz development. According to RNA-Seq data, in addition, expression levels of MYB-MIXTA-WD complex genes were not significantly different between DPL971 and DPL972, thus suggesting that Cotton_A_11941 may function at the downstream of the MYB-MIXTA-WD complex to participate in the control of fuzz development.

#### Author contribution statement

GS managed the project. GS, XF, HC and DZ designed the research. XF, HC, DZ, YZ, QW, KL, JA, QY, SL and XC performed the experiments. XF wrote the paper. All authors read and approved the final manuscript.

## Electronic supplementary material

Below is the link to the electronic supplementary material.

Supplementary material 1 (PDF 126 kb)

Supplementary material 2 (PDF 88 kb)

Supplementary material 3 (PDF 10 kb)

Supplementary material 4 (PDF 86 kb)

Supplementary material 5 (PDF 130 kb)

Supplementary material 6 (PDF 87 kb)

Supplementary material 7 (PDF 83 kb)

Supplementary material 8 (PDF 83 kb)
