# Supplementary material for: Fine mapping and identification of the fuzzless gene GaFzl in DPL972 (Gossypium arboreum)
Source: Theor Appl Genet. 2019 Apr 2;132(8):2169–79. doi: 10.1007/s00122-019-03330-3 (PMC6647196; doi:10.1007/s00122-019-03330-3)
Supplement: Supplementary file 1 — Supplementary material 1 (PDF 126 kb) [file 122_2019_3330_MOESM1_ESM.pdf]

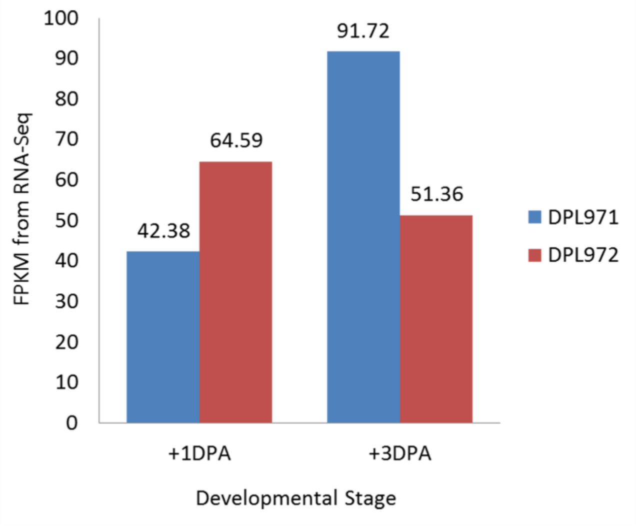

Fig. S1 Expression levels of *GaGL2* in DPL971 and DPL972 during different developmental stages. Data were obtained from RNA-Seq.
