# Supplementary material for: Fine mapping and identification of the fuzzless gene GaFzl in DPL972 (Gossypium arboreum)
Source: Theor Appl Genet. 2019 Apr 2;132(8):2169–79. doi: 10.1007/s00122-019-03330-3 (PMC6647196; doi:10.1007/s00122-019-03330-3)
Supplement: Supplementary file 2 — Supplementary material 2 (PDF 88 kb) [file 122_2019_3330_MOESM2_ESM.pdf]

TableS1 Information of BSA-seq data

Sample ID	Clean-Base	Q30(%)	Total_reads	Mapped(%)	Properly_mapped(%)	Ave_depth	Cov_ratio1X(%)
DPL971P1	53,736,668,700	90.96	358244458	97.98	81.8	31	64.59
DPL972P2	60,905,420,400	91.22	406036136	97.79	82.27	35	64.75
FuzzyPool1	60,910,934,100	90.82	406072894	98	81.43	35	64.85
FuzzlessPool2	53,419,518,900	91.23	356130126	97.7	81.56	30	64.54
