# Supplementary material for: Fine mapping and identification of the fuzzless gene GaFzl in DPL972 (Gossypium arboreum)
Source: Theor Appl Genet. 2019 Apr 2;132(8):2169–79. doi: 10.1007/s00122-019-03330-3 (PMC6647196; doi:10.1007/s00122-019-03330-3)
Supplement: Supplementary file 3 — Supplementary material 3 (PDF 10 kb) [file 122_2019_3330_MOESM3_ESM.pdf]

TableS2 Number of SNP detected by the BSA-seq

Type	R04vsR03	R06vsR05
INTERGENIC	598,506	455,600
INTRON	22,934	8,937
UPSTREAM	51,420	20,206
DOWNSTREAM	39,499	16,212
SPLICE_SITE_ACCEPTOR	23	10
SPLICE_SITE_DONOR	22	3
SPLICE_SITE_REGION	260	51
START_LOST	12	3
SYNONYMOUS_CODING	3,034	1,010
NON_SYNONYMOUS_CODING	4,314	1,353
SYNONYMOUS_STOP	4	2
STOP_GAINED	115	47
STOP_LOST	7	2
Other	142	115
Total	720,292	503,551

Note: R3: Fuzzy parent DPL971.

R4: Fuzzless parent DPL972.

R5: Bulked pool of 30 extreme fuzzy progenies.

R6: Bulked pool of 30 extreme fuzzless progenies.
