# Supplementary material for: Fine mapping and identification of the fuzzless gene GaFzl in DPL972 (Gossypium arboreum)
Source: Theor Appl Genet. 2019 Apr 2;132(8):2169–79. doi: 10.1007/s00122-019-03330-3 (PMC6647196; doi:10.1007/s00122-019-03330-3)
Supplement: Supplementary file 4 — Supplementary material 4 (PDF 86 kb) [file 122_2019_3330_MOESM4_ESM.pdf]

TableS3 Number of InDel detected by the BSA-seq

Type	R04vsR03	R06vsR05
INTERGENIC	81,013	60,307
INTRON	7,319	3,238
UPSTREAM	16,392	6,654
DOWNSTREAM	12,189	5,036
SPLICE_SITE_ACCEPTOR	8	5
SPLICE_SITE_DONOR	23	11
SPLICE_SITE_REGION	83	51
START_LOST	8	1
FRAME_SHIFT	372	180
CODON_DELETION	57	17
CODON_INSERTION	82	29
CODON_CHANGE_PLUS_CODON_DELETION	41	16
CODON_CHANGE_PLUS_CODON_INSERTION	18	16
STOP_GAINED	11	3
STOP_LOST	6	2
Other	26	22
Total	117,648	75,588

Note: R3: Fuzzy parent DPL971.

R4: Fuzzless parent DPL972.

R5: Bulked pool of 30 extreme fuzzy progenies.

R6: Bulked pool of 30 extreme fuzzless progenies.
