# Supplementary material for: Fine mapping and identification of the fuzzless gene GaFzl in DPL972 (Gossypium arboreum)
Source: Theor Appl Genet. 2019 Apr 2;132(8):2169–79. doi: 10.1007/s00122-019-03330-3 (PMC6647196; doi:10.1007/s00122-019-03330-3)
Supplement: Supplementary file 5 — Supplementary material 5 (PDF 130 kb) [file 122_2019_3330_MOESM5_ESM.pdf]

TableS4 Details of all designed markers in the fine mapping region of A08

Primer Name	Forward Primer (5'→3')	Reverse Primer (5'→3')	Product Length(bp)	Start position(bp)
SSR1	ATCACCTTGATTGGCACACA	TAAACAAGCCCATGCAAACA	209	7267
SSR2	GCATCAATTCCATGGTGTCTT	TGAAATTTCCACTAACTAGGATGAA	235	7906
SSR3	AGGGAAAGAAGCTGAGAGGG	TTAACTTGGGCCAGATACAAA	198	8899
SSR4	TTTGCTTAAGTCTATTTCGGTTGA	GGCTTGAGCTTAACCCGTTT	286	35886
SSR5	AATGACACTCTCCCGACGAC	TGAAGTGATGATATGGCACTTTG	169	36414
SSR6	TTCTCTGAGCTTGCTGGGAT	GGGACGTCAAGTAAATTAATCCT	278	61694
SSR7	AAACAAAGGTTGATGGCAAA	TTGGGTCGCTATCAATCTAAA	302	68265
SSR8	TTAAAGGTCCAACATCGCGT	TGCATGTATAGGTGAATCTTGAGG	251	78387
SSR9	GACCATGTTAATGACAACGATGA	TGCGCACTATCACATACCGT	182	79010
SSR10	ACCACATGACACATGGTAATTAGA	CCAATCAATGCCAGTCAACA	133	81310
SSR11	GAATTCCCTCACAGCTCCAA	GTCTCGCTGCGATTTCTAGG	329	82272
SSR12	GGGCCATTAAGCAATAGGGA	GGGAACCAACGAACTCAGTC	350	88280
SSR13	AAGCATAATAGCCCGTATGACAA	TTTAACGGCTTACTTCATGTTGA	181	88901
SSR14	CAATCAAGATCTGAACAAAGTCG	GCTCAAGCTCGACCTGATCT	259	103185
SSR15	CTCTCCCATTGCCATCACTT	TGGTTGGTATATGTAGTTGAGGGA	225	104037
SSR16	ATATGCATGGACGGATGGAT	CCGAGTTCGAAGCTGAAAGT	190	112969
SSR17	GTGCCAAGAACAAGGAGGAG	GTGCAATATTCATTGGACCG	296	114472
SSR18	CACGAGTGCTAATGGGACAA	ATCCAGGCAAATTGCGTACT	190	114727
SSR19	GCTAACACGCTCAGCAAGTG	TACTGGTGTCAACGATCCGA	293	115407
SSR20	TCGGATCGTTGACACCAGTA	ATCCTCCTCCTCCTCCTCCT	195	115718
SSR21	ACGCATAAACGAACTCGACA	AAATTCCAGGAAACTGCAAA	322	115733
SSR22	ATTCGGATATGCTCAGTGCC	TTGATGGTTAGTGCAATCCG	312	130308
SSR23	CCAATTGGAGAACACAAATTAAA	CCTTGGGATTTAGGCTTTCC	251	133736
SSR24	GTTTGGTGGCTTTGTTGGTT	CGAAGTTCAAACCTATATTCTCCAA	224	135323
SSR25	TTTGCGAGTACAATTATGTGAAA	CAAATCATGCTAGTAGTCCCTTTG	239	139953
SSR26	CTGTCGTATCCCTCCGTGTT	GATTTCAATGCCTGATCGCT	313	141729
SSR27	TCGATGTTATATCAATTAGGTCATTC	CGACTTTAGATCTCATATGGCGT	273	144282
SSR28	AAATGTCTCCGTTTCCATCG	CCTTCACCTGATTTCCCAAA	267	178822
SSR29	TTTGGGAAATCAGGTGAAGG	AGCTGAATGAGCAGCAAATG	264	179193
SSR30	TGCAAACCCACGTTATGTGT	GGTTAAAGGAGATGGGAGGG	250	189112
SSR31	GCGAATTTCTCTACCTCTGCAA	CACACATCAAACTTCTAATTGGAATAC	251	201766
SSR32	CTCATCCTCTCCATTGGCAT	ATCAATTCCCACATCAAGCC	283	216626
SSR33	AAGACGATGAAGAGCACGGT	GAAAGTCCACAAGCTGCTCC	205	217094
SSR34	AAATTGTTAAGTTTGCCCGC	CACTCTTGCTCCACCATCAA	241	217621
SSR35	TGTCATTTCTATCACAGCAGCA	TTGTTAGCAAGCATTCGTCG	282	219094
SSR36	TTGATAAATTCGATTGTTTGATTG	CCCTAGATTTGCATTGCATGA	313	234864
SSR37	GGGAGGGACTATGAGCAGTTC	AAGTTTAAGCCTCATCACATTCAA	126	235763
SSR38	ATGCTTGAACCCTGCCATTA	TCCCTTCAGCAACAATTGAA	262	251584
SSR39	AAGATTCAAAGTTTAGGACGTATGG	AAATCTTAAACTCTCGAAATGATTG	239	258303
SSR40	AGGGAAAGCGTTTCCAGTAA	ATGAATTCGGCCTGTCACTC	325	266237
SSR41	CGAAGAGGCTGAAACCCATA	ATGTTGCAGTTGGTGCTGAG	341	268367

SSR42	TGCATGCCTGATAATAAGCAA	CAGGGTCTGTTTGCAGAATTT	202	268972
SSR43	AGTGAAGAGTCGAATTTGCCA	AGCTGAATAGTTTGATGCATTATTT	298	276049
SSR44	TCACCCACTGTAATCAGTGCTC	TGGGTTTGAGTGTGCTGAAG	173	278986
SSR45	GTGCCAAACAACAATCTCCC	TGCATGTGGAGTTGAACCAT	187	280568
SSR46	TGTTTGATGCGAGTTCACCT	CATGTGAACCTTCTCTATTGCTTT	254	282267
SSR47	CCCAACATTAGCGGTGTTAG	GTGAAACGGAGGGAACTTCA	137	291474
SSR48	AGCCCAATTAGCCCACAAC	GATTTCAGGCTTTGTAGCCG	194	292801
SSR49	GGCTGAAATGGGTTAGGTCA	TTGAAAGTATTTACATCGGATAATGT	187	298359
SSR50	GGCTGAAATGGGTTAGGTCA	TTGAAAGTATTTACATGGGATAATGT	187	299425
SSR51	CACGGAAGTACACCCAACCT	GAGAAGCTACGAATGTCGGC	286	301593
SSR52	GGACTAAGACCATTATCTACTCCATAG	TTGAAAGTATTTACATGGGATAATGT	273	302988
SSR53	CGGCCAGTTGAATAGGAAAC	GGCTAAGGCTACTCAATCGC	298	303757
SSR54	GCCTTAATCCTCAAATTTCCC	CCCTAAGTGCCATGTGTGTG	250	317941
SSR55	CGAACACACAAAGACCCAAA	TGAAATTCAAATATGAACACCAAA	296	320488
SSR56	TGACCTTGACCCGGAAATAA	AAATCAAAGTTTATTATGCCCAAA	149	321626
SSR57	TGATATTGGCGTGGGAACTT	ATATTTGGCCCTCAACCTTT	287	322245
SSR58	TTGCAACTGTGGTTACTTACAGC	GGCATGAAGGAATGCAGATT	165	329336
SSR59	CAGTCAGATTGATTGGACTGAGA	TTGTGCACACCATCACTTGT	322	341123
SSR60	TTCCTGTCTGGGTTTGCTTT	CCGGAAGTTTAAGGTTGTTGC	281	342137
SSR61	CCGAACCACGTAAATCCTTG	TGAGCTACTCAATACTCAAACTCG	227	349626
SSR62	AGCCCATTTGATGCAATCTG	GCCAAATCGAAATCGAGAAC	180	357760
SSR63	CCAAGCTCAAGAGGGAATTG	CGAATGGAATCGATGAAGGA	339	361419
SSR64	TAGCTCATCGGTCCCAGTTT	GCACGCATGCATACCTACAT	228	366943
SSR65	CACTCGGCCCATAACTTGAT	CCGCCAACTTGGAAATCTAC	289	372200
SSR66	CGGGCCATGAACATCTCTAA	ACAGTAACAGGCGGTATCGG	344	374080
SSR67	ATGCCAGTAGGTGTTGGTCC	GTCCAGGATACGGTGTTGCT	337	375142
SSR68	ATGAGGAGGACGAGCAAGAA	CAGCCTGCTGAATCTGATGA	255	377194
SSR69	ATGAGGAGGACGAGCAAGAA	TGCCTTCTTCAGAATTGCCT	336	377214
SSR70	TTTATGATCCCTTTCACCCAA	GCATACAGCTGAATATATTAACACTCA	216	398847
SSR71	TGAAGGAAGAAGGGATTAATTTCA	AGAGCCAAAGGGTAGTGGGT	154	419165
SSR72	TCGATGGTGTATTTGGCTCA	ATGCTTTGTTCCACAGCACA	299	424052
SSR73	ATTGTCGGTACAGGAGGACG	TCCTTGTGCAAGAAGACGTG	221	424456
SSR74	AAATCCAAGAGAATTGAGTGATTAAA	GTTGCTACTTGCCACGTTGA	197	425765
SSR75	CATGTTAGTCGGTTGGTGACA	TGCAGATCACAGAGAATGGC	302	437510
SSR76	TGCCATTCTCTGTGATCTGC	ATGATTCCAACCCGATTCAA	176	437770
SSR77	CTGATTCTCAAGTGGCACGA	TTGCAAATTCATCTCCCTTTG	156	440278
SSR78	GGCACTTGGCATTGTTTCTT	ACGGCAAAGTTGACGGAATA	223	444245
SSR79	CCACAATCAAAGTTTAATGCGTA	GAGCTCTAGTTTAGTCGGGTGG	225	446768
SSR80	TCAACTTCAATGCACGAGGA	GCAAACAGCTCTGTGAACGA	125	455339
SSR81	CTGAAACCCTCATTTGCAGC	GGGTCTCTTCGCCGTAGTC	157	459808
SSR82	CCTTCCATGCATATTGGAAA	CAAAGCACCCAATTTCAAGG	283	469010
SSR83	CACCTTGAAATTGGGTGCTT	GCTTGGTGACCCTCTAGCTG	191	469261
SSR84	AGCTAGAGGGTCACCAAGCA	CATCCCACTAGCAGCATTGA	162	469435
SSR85	TGTTAATCATGTGATTTATTGAGTGTG	CATGAACTACCACGCGGTTT	257	470082

SSR86	TGTTGTTAAATTGCTTACTTGGGA	TCGATACCTACCAAGGTTTGAA	310	485966
SSR87	TTATTATCCGCCCAAACAGC	AACTTGGCAGGTGGATTGAT	325	486744
SSR88	TCCTATTTGCTGGCGAGATT	AAGTGGCCTGACCCATACAC	316	490174
SSR89	TGAACGTATACGAAGATTTGGTT	AAGTGGCCTGACCCATACAC	253	490296
SSR90	AATGCCTTACCTTTGAAATGC	TGGACTTGTTCTTTCTTGTTGC	136	491476
SSR91	AACAGGAGCATTGATGCAAA	GCTGCTGAAGCTGAGGCTAT	306	498723
SSR92	GTATCTGCAAGGGTGGAGGA	AAGATACGCAATGGCAAAGC	203	520481
SSR93	TTTGAGTTTAAATTGAACGGATTT	TCTTGGTGGTGGAGTTCACA	160	544219
SSR94	TGTGAACTCCACCACCAAGA	TTCACTACCATCATTCGGGA	259	544357
SSR95	TGTGAACTCCACCACCAAGA	TTCACTACCATCATTCGGGA	259	544421
SSR96	GGGCAAACTGCAATACGAGT	AACCAATCAGCATAGCCCAC	275	546417
SSR97	TTGTTTCGCAACGAATTAAATG	TTAACCTTACTCCTTCGAACCAT	237	547865
SSR98	AATTGTGGGATCTGCTGCAT	TGGCGTGGACATGTACCTTA	323	551918
InDel1	GGCTGCCACCCCCC	CCTCACATTTAACGTCAAGCTCAC	356	76294
InDel2	CCAAACATCCATGTGGACCAATTTG	GGTAATAACTGTTCTTTATGAGCCCTTTTTC	360	501736
InDel3	CATCAACGTGGCAAGTAGCAACA	CCCAGGGGCAAAAAAAATTTGGG	372	525615
InDel4	GCCCCTCCAGATTTGCCTC	GGTCCAAATTGTCTAAAATAAATCCGTAGAGAC	565	531453
InDel5	CGAATCCTGAACCCCAAACCTAAAC	CATCACCATGGCAACAACTCC	376	532619
InDel6	CGCGAGGACTAAAATTTGAAAGTTTGGA	GGTAAGGATTGGGGCATTAACTGG	178	534201
InDel7	CCATAATTAGAGTTTCACGTGTATAATTGTACC	GAGTTTTGTGTCACCACTCAATCG	256	534829
InDel8	GGCTTGTGCAGCCATTGTG	TCGTGCATTAAAAAGAACATGCTAGG	377	640157
InDel9	GATTTCCCTTTCATCAAATATTCTATGTTAGCG	ACTCAAATACTTAGTATACCATATTAGCCTCTTTC	536	650514
InDel10	AGTAGGTGCTACAAAATCAGCTCC	CTGTTTTAGCATTTTGGGTGCTCTTTTC	327	664027
InDel11	CACAACATTTAGTAAATAGAAATTTTGGGCAATCC	GGTTATTGACTTGAAGAGGGGGG	252	666443

Note: markers in highlight are polymorphic between two mapping parents.
