# Supplementary material for: Fine mapping and identification of the fuzzless gene GaFzl in DPL972 (Gossypium arboreum)
Source: Theor Appl Genet. 2019 Apr 2;132(8):2169–79. doi: 10.1007/s00122-019-03330-3 (PMC6647196; doi:10.1007/s00122-019-03330-3)
Supplement: Supplementary file 6 — Supplementary material 6 (PDF 87 kb) [file 122_2019_3330_MOESM6_ESM.pdf]

TableS5 Information of RNA-seq data

| Sample | CleanBases (bp) | CleanQ30(%) | Total reads | Read mapping rate | Multiple mapping rate |
|---|---|---|---|---|---|
| DPL971+1R1 | 7,168,622,700 | 95.38 | 23895409 | 91% | 5% |
| DPL971+1R2 | 6,412,138,500 | 95.4 | 25321633 | 91% | 9% |
| DPL971+3R1 | 7,571,760,300 | 95.31 | 25239201 | 91% | 6% |
| DPL971+3R2 | 7,265,123,100 | 95.46 | 23930032 | 91% | 5% |
| DPL971+5R1 | 7,042,378,500 | 95.27 | 23474595 | 91% | 5% |
| DPL971+5R2 | 8,017,725,900 | 95.28 | 27254966 | 91% | 6% |
| DPL972+1R1 | 7,596,489,900 | 95.41 | 21373795 | 91% | 7% |
| DPL972+1R2 | 8,608,806,300 | 95.46 | 28696021 | 91% | 7% |
| DPL972+3R1 | 7,179,009,600 | 95.25 | 24217077 | 91% | 4% |
| DPL972+3R2 | 7,619,923,500 | 95.24 | 25399745 | 91% | 5% |
| DPL972+5R1 | 8,176,489,800 | 95.51 | 26725753 | 92% | 6% |
| DPL972+5R2 | 7,619,780,400 | 95.35 | 25399268 | 91% | 7% |
