# Supplementary material for: Fine mapping and identification of the fuzzless gene GaFzl in DPL972 (Gossypium arboreum)
Source: Theor Appl Genet. 2019 Apr 2;132(8):2169–79. doi: 10.1007/s00122-019-03330-3 (PMC6647196; doi:10.1007/s00122-019-03330-3)
Supplement: Supplementary file 7 — Supplementary material 7 (PDF 83 kb) [file 122_2019_3330_MOESM7_ESM.pdf]

TableS6 Sequence information about amplification primers of genes and promoters

Primer Name	Product Length(bp)	ForwardPrimer (5'→3')	ReversePrimer (5'→3')
11941	264	ATGTCAGTTTCTCCCCTGGAAATATCG	TCAATTGCTTGCCCATTTAGCAGTC
11941P	2000	TCCCAGGTCACTAACAATATTGTCG	GACATGCTTGGTGACCCTCTAG
11942	843	ATGGGTTTTGAAGATTCAGATAGGGTATC	TTAATAAACCTCTAATCCAAATAATACTCCGGCA
11942P	2000	CTATGACCATGATTACGCCAAGCTTGTCTATTTTGGTCATTAACGGGCTAAC	ACCACCCGGGGATCCTCTAGATATTGGAAAATCCAAGAAATTACAACAAAAAAAAAGAGA
