# Supplementary material for: Fine mapping and identification of the fuzzless gene GaFzl in DPL972 (Gossypium arboreum)
Source: Theor Appl Genet. 2019 Apr 2;132(8):2169–79. doi: 10.1007/s00122-019-03330-3 (PMC6647196; doi:10.1007/s00122-019-03330-3)
Supplement: Supplementary file 8 — Supplementary material 8 (PDF 83 kb) [file 122_2019_3330_MOESM8_ESM.pdf]

TableS7 Sequence information of qRT-PCR primers

Primer Name	Product Length(bp)	ForwardPrimer (5'→3')	ReversePrimer (5'→3')
q11941-2	137	GTCTTCGGAACCCGGAGATG	TCCCACTAGCAGCATTGACG
q11941-3	114	TGGGTGCTTTGAAGGCTTGT	CTGACATGCTTGGTGACCCT
q11942-1	126	GGAGTTCGAGTTGATCAGCCA	TTTGAGTACCGTTGTACGGC
q11942-2	133	TCCTGACCCGACGAGATTTG	CCCACCATACAAGCTGTTACAA
